# SATB1, genomic instability and Gleason grading constitute a novel risk score for prostate cancer

**DOI:** 10.1038/s41598-021-03702-0

**Published:** 2021-12-27

**Authors:** Christoph Dumke, Timo Gemoll, Martina Oberländer, Sandra Freitag-Wolf, Christoph Thorns, Axel Glaessgen, Rinse Klooster, Silvère M. van der Maarel, Jerker Widengren, Christian Doehn, Gert Auer, Jens K. Habermann

**Affiliations:** 1 Section for Translational Surgical Oncology and Biobanking, Department of Surgery, University of Lübeck and University Hospital Schleswig-Holstein, Campus Lübeck, Ratzeburger Allee 160, 23538 Lübeck, Germany; 2 Interdisciplinary Center for Biobanking-Lübeck (ICB-L), University of Lübeck, Lübeck, Germany; 3 Institute of Medical Informatics and Statistics, University Hospital Schleswig-Holstein, Campus Kiel, Kiel, Germany; 4 Institute of Pathology, University of Lübeck and University Hospital Schleswig-Holstein, Campus Lübeck, Lübeck, Germany; 5 Department of Clinical Pathology and Cytology, Unilabs AB, Stockholm, Sweden; 6 Department of Human Genetics, Leiden University Medical Center, Leiden, The Netherlands; 7 Department of Applied Physics, Royal Institute of Technology (KTH), Stockholm, Sweden; 8 Urologikum Lübeck, Lübeck, Germany; 9 Department of Oncology-Pathology, Karolinska Institutet, Stockholm, Sweden

**Keywords:** Cancer, Urology

## Abstract

Current prostate cancer risk classifications rely on clinicopathological parameters resulting in uncertainties for prognostication. To improve individual risk stratification, we examined the predictive value of selected proteins with respect to tumor heterogeneity and genomic instability. We assessed the degree of genomic instability in 50 radical prostatectomy specimens by DNA-Image-Cytometry and evaluated protein expression in related 199 tissue-microarray (TMA) cores. Immunohistochemical data of SATB1, SPIN1, TPM4, VIME and TBB5 were correlated with the degree of genomic instability, established clinical risk factors and overall survival. Genomic instability was associated with a GS ≥ 7 (*p* = 0.001) and worse overall survival (*p* = 0.008). A positive SATB1 expression was associated with a GS ≤ 6 (*p* = 0.040), genomic stability (*p* = 0.027), and was a predictor for increased overall survival (*p* = 0.023). High expression of SPIN1 was also associated with longer overall survival (*p* = 0.048) and lower preoperative PSA-values (*p* = 0.047). The combination of SATB1 expression, genomic instability, and GS lead to a novel Prostate Cancer Prediction Score (PCP-Score) which outperforms the current D’Amico et al. stratification for predicting overall survival. Low SATB1 expression, genomic instability and GS ≥ 7 were identified as markers for poor prognosis. Their combination overcomes current clinical risk stratification regimes.

## Introduction

With 1,276,106 new cases in 2018, prostate cancer is the second most frequent malignant disease among males worldwide^1^. Further, it remains the sixth leading cause of malignancy associated death^1^. In clinical practice, the classification according to D’Amico containing preoperative PSA serum level, biopsy-based Gleason Score (GS) and clinical tumor stage is routinely used to distinguish between high, intermediate and low risk for PSA recurrence after radical prostatectomy (RPE), external radiotherapy or brachytherapy^2^. More contemporary validation studies of the D’Amico classification confirmed its ability to predict patients’ risk of PSA recurrence after RPE and death from prostate cancer^3, 4^. However, preoperatively classified low risk patients showed heterogenous histology and were frequently upgraded or upstaged at final examination after RPE^5^. Furthermore, intermediate and high risk patients still remain heterogenous groups with different prognosis depending on whether clinical tumor stage or biopsy-based GS are considered^6^. Thus, this classification can fail resulting in possible over- or undertreatment^6, 7^. Improved prognostication for individualized therapy can be achieved by assessment of intra-tumor heterogeneity on the molecular level which is underrepresented in D’Amico’s classification but yet an important trait of solid tumors and associated with genomic instability^8, 9^. Consequently, there is an unmet opportunity to account and address tumor biology more precisely. For this purpose many genes and their products have been evaluated as potential biomarkers for prostate cancer^10^. Our literature review revealed special-AT-rich-protein-1 (SATB1), Spindlin-1 (SPIN1), Vimentin (VIME), β-Tubulin (TBB5), and Tropomyosin-4 (TPM4) to be the most promising biomarkers^11–15^.

SATB1 is a nuclear chromatin organizer regulating the expression of genes involved in cell differentiation and apoptosis^16^. SATB1 seems to be upregulated in malignant tumors of the breast, colon, and bladder cancer causing aggressive tumor growth and limited prognosis^17–19^. SATB1 levels also appear to be increased in prostate cancer with implications for tumor progression and metastasis through Epithelial-Mesenchymal-Transition (EMT)^12, 20^.

SPIN1 represents a nuclear factor that associates with the spindle apparatus during meiosis and is highly expressed in early embryonic tissues^21^. Moreover, its levels were found to be increased in ovarian cancer and liposarcoma^13, 22^.

TPM4, VIME, and TBB5 are dynamic parts of the cytoskeleton and play an important role in cell motility and metastasis^11, 23, 24^. Despite their tumor biology functions, neither SATB1, SPIN1, VIME, TBB5 nor TPM4 are yet being used in routine risk assessment for prostate cancer. Therefore, we examined their protein expression at multiple tumor sites to address potential intra-tumor heterogeneity^8, 25^ and correlated their protein expression to the degree of genomic instability, established clinical risk factors, as well as overall survival.

## Material and methods

### Patient collective

Formalin fixed and paraffin embedded prostate cancers were obtained from 50 patients that had undergone radical prostatectomy at the University Hospital Schleswig–Holstein, Campus Lübeck between 1989 and 1992. After histological confirmation, all operations were carried out using the open-surgery technique with diagnostic lymphadenectomy. In case of PSA values ≥ 10 ng/ml a preoperative bone scintigraphy was performed in order to exclude bone metastases. Neoadjuvant chemo- and/or antihormonal therapy were not administered. Postoperative treatment was performed in an outpatient setting and is—retrospectively—not retrievable. However, it can be envisioned that patients at that time (1989–1992) were treated comparably. The median follow-up was 15 years (range 1–22 years). Survival data was obtained from the residences’ registration offices. All clinical and pathological data are summarized in Table 1. Risk groups were assessed as follows: low risk (T1-T2a, Gleason Score (GS) ≤ 6, and PSA < 10 ng/ml), intermediate risk (T2b-T2c or GS 7 or PSA 10–20 ng/ml), and high risk (≥ T3 or GS 8–10 or PSA > 20 ng/ml). However, despite D’Amico’s original classification being based on preoperative biopsy-based GS and clinical stage, only pathological GS and pathological stage were available for this study. The GS was retrospectively re-evaluated by one senior pathologist (C.T.) according to the recommendations of the International Society of Urological Pathology 2005 using 4 µm Hematoxylin and Eosin (HE) stained tissue sections^26^. The study was conducted according to the guidelines of the Declaration of Helsinki. All prostate cancer samples were collected within clinical routine diagnostics at the University Hospital Schleswig–Holstein in Lübeck, Germany, between 1989 and 1992. At that time, no informed consent for research purposes was obtained. The study was approved by the local Ethics Committee of the University of Lübeck which also approved the use of anonymized samples and anonymized data for this retrospective study without contacting the patients (#08–011 and #20-508).

**Table 1:** Patients data.

Parameter	n	%
**Age (median)**		
≤ 63	27	54
> 63	23	46
**Tumor stage**		
≤ pT2b	13	26
pT3–pT4	35	70
Not available	2	4
**Lymph node stage**		
pN0	47	94
pN1	1	2
Not available	2	4
**Metastasis**		
cM0	41	82
cM1	0	0
Not available	9	18
**Gleason Score**		
4–6	24	48
7	16	32
8–10	10	20
**Preoperative PSA [ng/ml]**		
< 10	12	24
10–20	16	32
> 20	11	22
Not available	11	22
**Postoperative survival after 22 years**		
Alive	15	30
Dead	33	66
Not available	2	4
**Genomic stability**		
Stable	32	64
Instable	16	32
Not available	2	4

### Tissue microarray (TMA)

The tissue microarray was constructed as described^27^. Tissue areas containing cancer and benign cells were marked by one senior pathologist (C.T.). Subsequently, 1.5 mm cores of both, cancer and benign tissue from each patient, were received by a TMArrayer™ (Pathology Devices, Inc. Westminster, USA) and built into a new custom-made TMA. Based on previous reports on optimal tissue representativity for heterogenous tumors^25^, we decided to obtain four tissue cores from different sites of the marked tumor areas and one core from benign tissue for each patient. Thus, 199 tumor and 48 benign tissue cores were distributed onto two TMA blocks. Benign tissue was not available for two patients. Tissue integrity and histology were examined after HE-staining.

### Assessing the degree of genomic instability

Genomic instability was assessed by DNA image cytometry using 8 µm Feulgen stained whole tissue sections of each tumor. Two patients lacked enough material for DNA image cytometry. Cell selection criteria, quantitative measurement of nuclear DNA content, and internal standardization were based on methods described^28^. Detection of representative tumor cell nuclei on Feulgen stained tissue sections was performed using marked tumor areas on corresponding HE slides using an ICM imaging system (Ahrens ICM Cytometry System, Hamburg, Germany). The DNA content of at least 100 nuclei per specimen was quantitatively measured and expressed in relation to the DNA content of lymphocytes as reference which was given the value 2c reflecting a physiological diploid DNA content. The resulting DNA histograms were classified according to Auer et al.^28, 29^ (Supplemental Data 1, Figure S1).

### Immunohistochemistry

After immunohistochemical staining (Supplemental Data 2), scoring was performed semiquantitatively by one independent senior pathologist (A.G.) according to the immunoreactive Score (IRS)^30^ (Figure S2–S5). For VIME, an alternative score regarding the percentage of positive tumor cells was used: Score 0—negative staining of all tumor cells; Score 1—up to 20% stained tumor cells; Score 2—21 to 50% stained tumor cells; Score 3—more than 50% stained tumor cells (Figure S6). A high expression was defined as IRS 6–12 for SATB1 and TPM4, and as IRS 9–12 for SPIN1 and TBB5. For VIME a tissue core was assessed for high expression when showing a Score of 1–3. A tumor was designated as positive when at least one of the four obtained tissue cores showed a high expression for the evaluated protein, whereas a low or negative expression in all four tissue cores defined a tumor as negative. To determine intra-tumor heterogeneity, we additionally evaluated all TMA tissue cores per patient. Homogenous expression was declared if all cores per patient showed either only high or only low protein expression. In contrast, in-homogenous expression was assessed with two independent approaches: we (i) accounted for the number of tissue cores with a high protein expression and (ii) considered the expression of only one randomly chosen tissue core (random).

### Statistics

Statistical analysis was performed using SPSS version 21 (IBM Corporation, Somer, NY, USA). Continuous variables were dichotomized or categorized based on clinical and pathological parameters. Associations between categorical variables were assessed by Fisher’s exact test, and two-sided *P*-values < 0.05 were considered to be statistically significant.

For the analysis of the survival times, Kaplan Meier estimators, log-rank tests and age-adjusted cox regression models were used to identify potential risk factors. Their impact upon patients’ mean overall survival (OS) was expressed as hazard ratio (HR) provided with 95% confidence intervals (CI). For multivariable analysis, we used the Cox proportional hazard model and included the established prognostic factors tumor stage, preoperative PSA, Gleason Score, as well as prognostic factors and their combinations that were significant in univariable Cox regression (SATB1 positive, SATB1 ≥ 2cores, GS ≥ 7 or genomic instability, GS ≥ 7 or SATB1 negative, GS ≥ 7 or SATB1 ≤ 1core, genomic instability or SATB1 negative, genomic instability or SATB1 ≤ 1 core, GS/genomic instability/SATB1 ≤ 1 core; Supplemental Table S1) as well as the new PCP-Score. All possible predictors were selected on the likelihood ratio criteria in the following backward selection. Furthermore, the prediction of the new Score (PCP) was analyzed in the strata with a low and a high number of SATB1 expressing tissue cores (≤ 1 vs. ≥ 2) in order to investigate possible interactions. Beyond this, a model with and without genomic instability was fitted for a comparison of the prediction performance. For all models the proportional hazard assumption was checked using weighted residuals and none of the prognostic factors were found to violate this assumption.

### Ethics approval and consent to participate

Approval from the local ethics committee of the University of Lübeck (#08-011 and #20-508).

## Results

### Associations of clinical parameters with survival

The mean overall survival (OS) of the entire patient collective was 14.3 years (CI, 12.3–16.2).

A low Gleason Score (GS) of 4–6 was found in 24 cases (48%), whereas 26 (52%) showed a GS of 7 or higher. Patients with a GS of 4–6 had a longer OS of 17.9 years (CI, 15.9–20) compared to 11 years (CI, 8.5–13.6) for patients with a GS of 7–10 (*p* = 0.002, Table S1). Furthermore, the GS was a significant predictor of OS in univariable cox regression analysis (age-adjusted HR 3.394, CI 1.615–7.132, *p* = 0.001, Table S1). Neither pathological stage nor preoperative PSA value showed a significant association with OS (Table S1).

### Detecting the degree of genomic instability

Overall, 16 of 48 (33.3%) tumors showed nuclear DNA aneuploidy denoting genomic instability. Patients with euploid, genomically stable tumor cell populations had a longer OS with 16.5 years (CI, 14.3–18.8) compared to aneuploid, genomically instable tumors with 10.8 years (CI, 7.6–14) (*p* = 0.006, Table S1, Figure S7). Furthermore, we could show that genomic instability was significantly associated with a higher tumor grade: 14 tumors (56%) with GS 7–10 showed genomic instability in contrast to only 2 (8.7%) of GS 4–6 carcinomas (*p* = 0.001, Table S2). Patients with genomically instable tumor cell populations showed a 2.7-fold increased mortality risk in univariable analysis (age-adjusted HR 2.672, CI 1.294–5.515, *p* = 0.008, Table S1).

### Determining intra-tumor heterogeneity by protein expression

**Figure 1:**
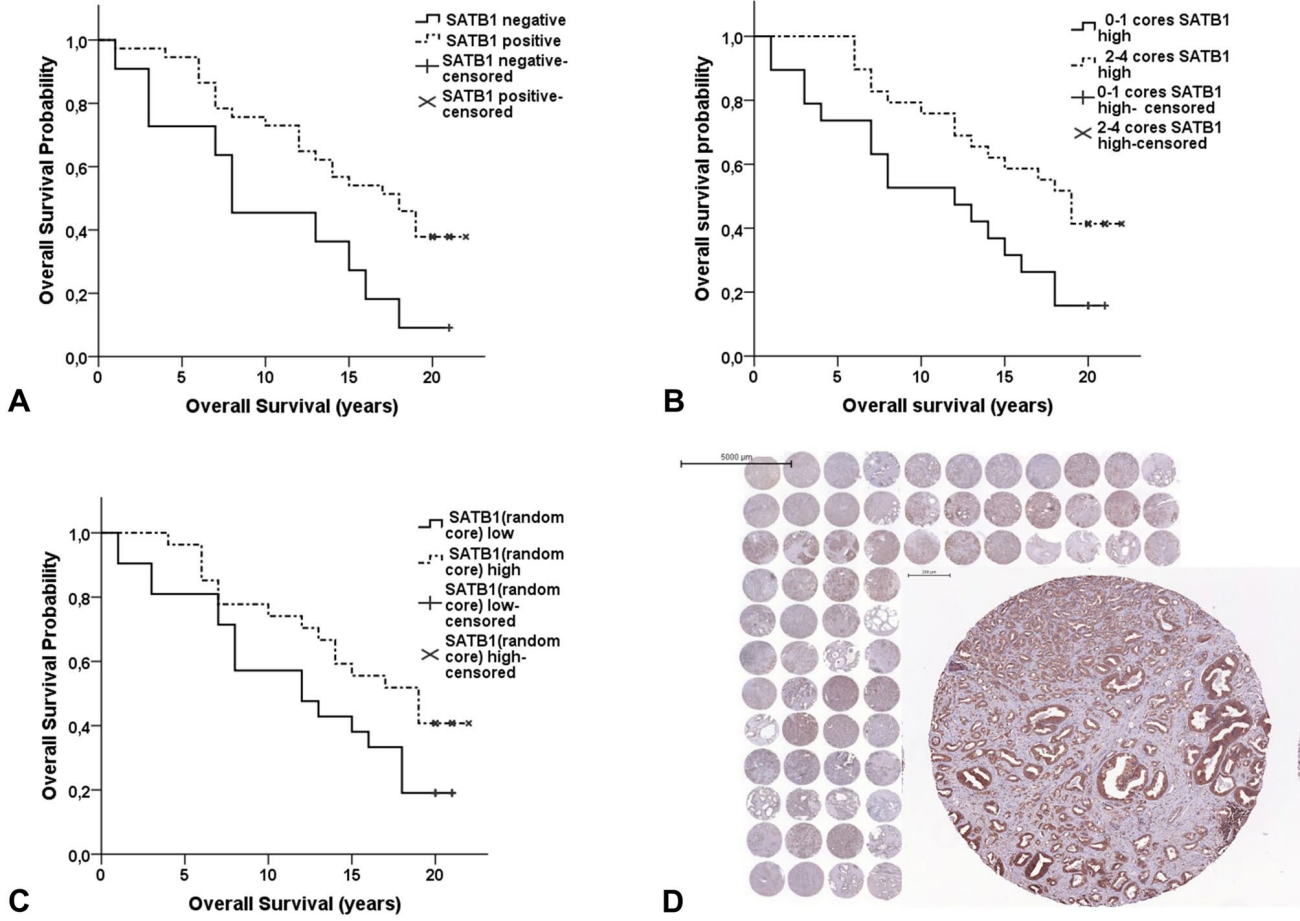
Overall Survival stratified by (**A**) at least one tissue core with a high SATB1 expression per tumor (SATB1 positive), *p* = 0.02; (**B**) the number of tissue cores with a high SATB1 expression per tumor, *p* = 0.018; (**C**) and the SATB1 expression in one randomly chosen tissue core per tumor, *p* = 0.068; (**D**) TMA slide with SATB1 stained tissue cores (one TMA tissue core enlarged). *P*-values for the log-rank test.

Extensive heterogeneity of up to 48% was observed for the expression of SATB1 and TBB5 (Table 2). For SATB1, nuclear and cytoplasmic immunoreactivity occurred in epithelial tumor cells or normal epithelium of the prostate but not in stromal tissue cells (Figure S2). While 39 (78%) carcinomas were SATB1 positive, only 11 (22.9%) benign tissue samples were strongly immunoreactive for SATB1 (*p* < 0.001, Table 3). SATB1 positive patients had a longer OS of 15.5 years (CI, 13.3–17.6) compared to 10.3 years (CI, 6.5–14) for SATB1 negative ones (*p* = 0.020, Table S1, Fig. 1A). In line, a positive SATB1 expression was associated with a GS of 4–6 (*p* = 0.040, Table 3) and genomic stability (*p* = 0.027, Table 3).

**Table 2:** Tumor heterogeneity in protein expression.

No. of patients (%)	SATB1 N = 50	SPIN1 N = 50	TBB5 N = 50	VIME N = 50	TPM4 N = 50
Homogeneous expression 4 of 4 tissue cores high or low	26 (52%)	39 (78%)	26 (52%)	43 (86%)	43 (46%)
Heterogeneous expression in total	24 (48%)	11 (22%)	24 (48%)	7 (14%)	7 (14%)
3 of 4 tissue cores high	7	0	5	0	1
2 of 4 tissue cores high	8	2	5	0	0
1 of 4 tissue cores high	9	9	14	7	6

**Table 3:** Significant associations between proteins and clinicopathological parameters.

Parameter	Gleason Score [N = 50]		P*	Tumor stage [N = 48]		P*	PSA (ng/ml) [N = 39]		P*	Ploidy [N = 48]		P*	Histopathology [N = 98]**		P*
	4–6	7–10		≤ T2b	≥ T3		≤ 10	> 10		Genomically stable	Genomically instable		Benign	Cancer	
**SATB1**															
Positive	**22** **(91.7%)**	**17** **(65.4%)**	**0.040**	10 (76.9%)	27 (77.1%)	1.000	12 (85.7%)	17 (68%)	0.279	**28** **(87.5%)**	**9** **(56.2%)**	**0.027**	**11** **(22.9%)**	**39** **(78%)**	** < 0.001**
Negative	**2** **(8.3%)**	**9** **(34.6%)**		3 (23.1%)	8 (22.9%)		2 (14.3%)	8 (32%)		**4** **(12.5%)**	**7** **(43.8%)**		**37** **(77.1%)**	**11** **(22%)**	
**SPIN1**															
Positive	7 (29.2%)	4 (15.4%)	0.314	3 (23.1%)	8 (22.9%)	1.000	**6** **(42.9%)**	**3** **(12%)**	**0.047**	8 (25%)	3 (18.7%)	0.729	**3** **(6.4%)**	**11** **(22%)**	**0.042**
Negative	17 (70.8%)	22 (84.6%)		10 (76.9%)	27 (77.1%)		**8** **(57.1%)**	**22** **(88%)**		24 (75%)	13 (81.3%)		**44** **(93.6%)**	**39** **(78%)**	
**TPM4**															
Positive	7 (29.2%)	2 (7.7%)	0.069	3 (23.1%)	6 (17.1%)	0.687	3 (21.4%)	4 (16%)	0.686	7 (21.9%)	2 (12.5%)	0.697	**1** **(2.1%)**	**9** **(18%)**	**0.016**
Negative	17 (70.8%)	24 (92.3%)		10 (76.9%)	29 (82.9%)		11 (78.6%)	21 (84%)		25 (78.1%)	14 (87.5%)		**47** **(97.9%)**	**41** **(82%)**	
**VIME**															
Positive	3 (12.5%)	7 (26.9%)	0.294	2 (15.4%)	7 (20%)	1.000	5 (35.7%)	2 (8%)	0.075	5 (15.6%)	4 (25%)	0.457	**20** **(42.5%)**	**10** **(20%)**	**0.027**
Negative	21 (87.5%)	19 (73.1%)		11 (84.6%)	28 (80%)		9 (64.3%)	23 (92%)		27 (84.4%)	12 (75%)		**27** **(57.5%)**	**40** **(80%)**	
**TBB5**															
Positive	16 (66.7%)	11 (42.3%)	0.098	7 (53.8%)	19 (54.3%)	1.000	5 (35.7%)	15 (60%)	0.191	**22** **(68.7%)**	**4** **(25%)**	**0.006**	**10** **(20.8%)**	**27** **(54%)**	**0.001**
Negative	8 (33.3%)	15 (57.7%)		6 (56.2%)	16 (45.7%)		9 (64.3%)	10 (40%)		**10** **(31.3%)**	**12** **(75%)**		**38** **(79.2%)**	**23** **(46%)**	

*P-Value for Fisher’s Exact Test.

**Because of tissue loss during staining N = 97 for SPIN1 and VIME.

Significant *p*-values are highlighted in bold.

Further, patients with ≥ 2 tissue cores with high SATB1 expression had an OS of 16.3 years (CI, 14.1–18.5) compared to 11.1 years for patients with only ≤ 1 high SATB1 tissue cores (CI, 8.0–14.2) (*p* = 0.018, Table S1, Fig. 1B). Interestingly, age adjusted cox regression analysis showed that SATB1 positive patients (at least one tissue core with a high SATB1 expression) and patients with ≥ 2 tissue cores with high SATB1 expression had a significantly lower risk of dying during the observation period (age-adjusted HR 0.413, CI 0.192–0.886, *p* = 0.023 & HR 0.479, CI 0.238–0.965, *p* = 0.039, Table S1). No significant results were obtained when SATB1 expression was evaluated in only one tissue core selected at random from each tumor (*p* = 0.068, Fig. 1C,D).

For SPIN1, TBB5, VIME and TPM4, significance in their cytoplasmic staining could be detected between cancer and normal epithelium cells: while VIME had higher protein levels in benign prostate tissue (*p* = 0.027, Table 3), SPIN1, TBB5 and TPM4 showed higher expression in cancer tissue (*p* = 0.042, *p* = 0.001 and *p* = 0.016, respectively, Table 3). Furthermore, patients with genomically stable tumor cell populations had a higher TBB5 expression (*p* = 0.006, Table 3). However, only SPIN1 positivity was associated with a low preoperative PSA value (Table 3) and a longer OS (Figure S7), but none of these proteins were significant predictors of OS in univariable cox regression analysis.

### Developing a new prediction score

**Figure 2:**
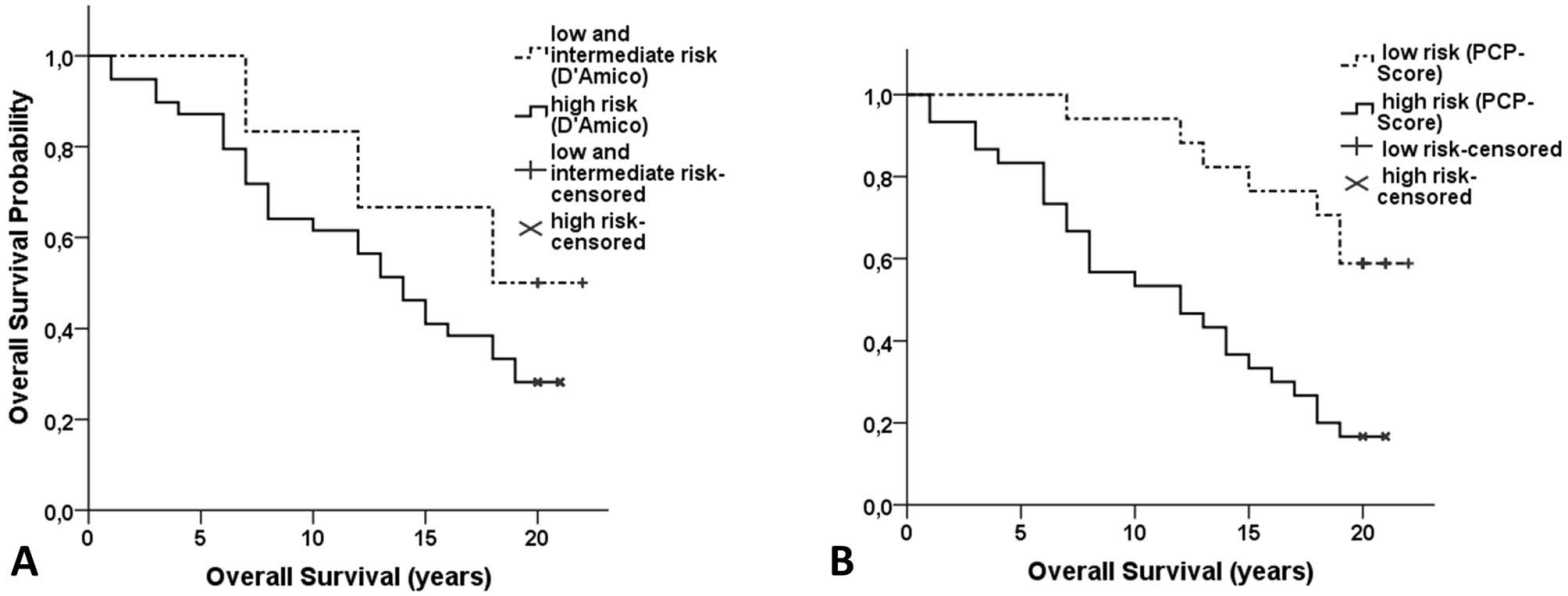
Overall Survival stratified according to (**A**) D’Amico classification: low and intermediate risk (GS ≤ 7 and PSA ≤ 20 ng/ml and ≤ T2b) versus high risk (GS > 7 or PSA > 20 ng/ml or ≥ T3), *p* = 0.285 and (**B**) new PCP-Score: low risk (GS ≤ 6 and genomic stability and high SATB1 expression in at least one tissue core) versus high risk (GS ≥ 7 or genomic instability or low/negative SATB1 expression in all four obtained cores), *p* = 0.001; *P*-values for the log-rank test.

Grouping our patients according to D’Amico et al., six patients were at low or intermediate risk, whereas 39 were at high risk. Interestingly, this classification failed in predicting postoperative OS (*p* = 0.285, Fig. 2A).

**Figure 3:**
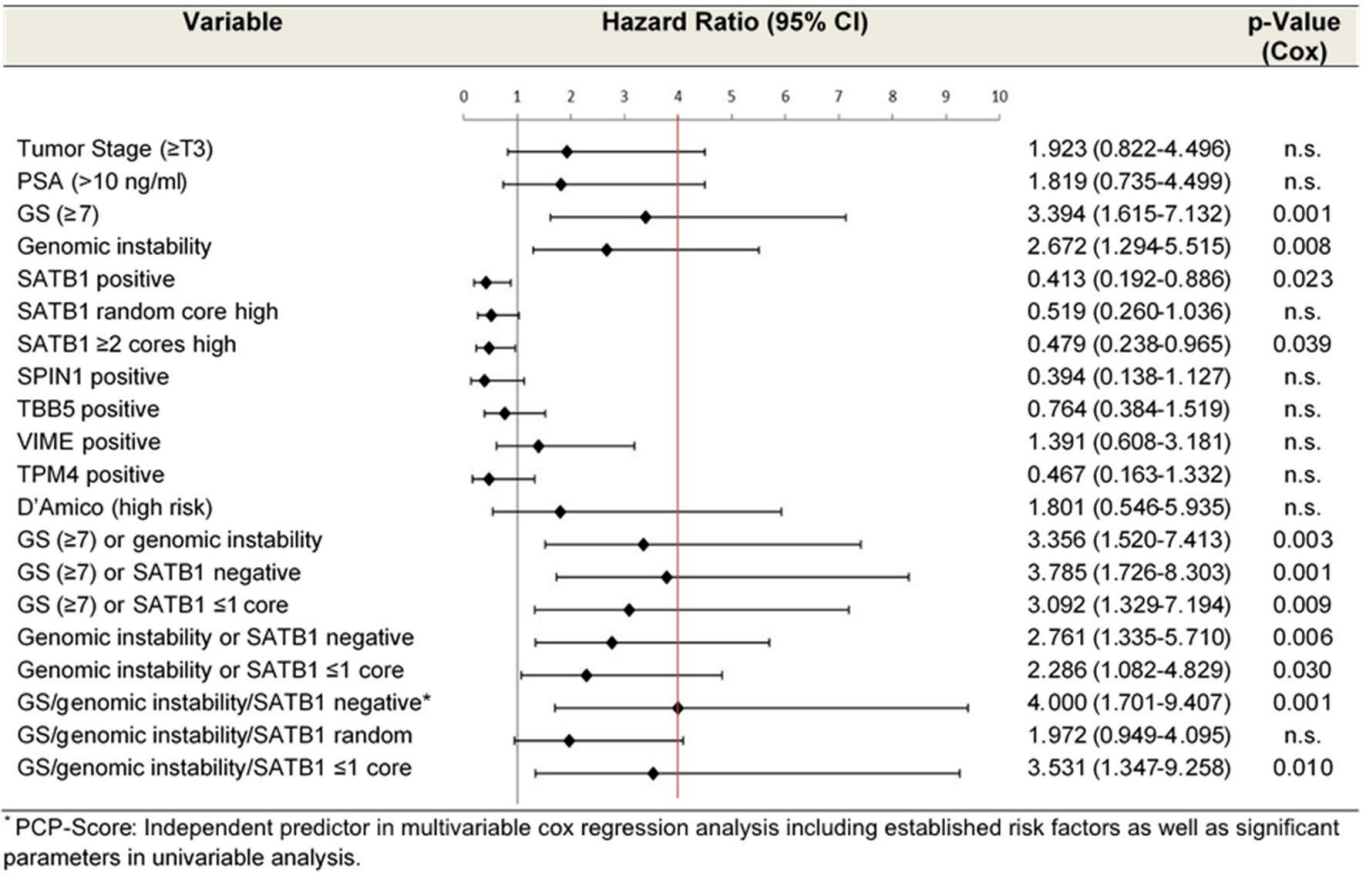
Age-adjusted hazard ratios of univariable cox regression analysis for all investigated parameters including combined parameters and the new Prostate Cancer Prediction Score (PCP). Positive = at least one tissue core with a high protein expression. SATB1 ≥ 2 cores =  ≥ 2 tissue cores with a high protein expression. SATB1 ≤ 1 core =  ≤ 1 tissue core with a high SATB1 expression. SATB1 random = high protein expression in only one randomly chosen tissue core. Red line: Hazard Ratio of new PCP-Score.

Our findings prompted us to combine all risk factors with a strong impact on OS, namely SATB1 expression, degree of genomic instability and GS for a new Prostate Cancer Prediction Score (PCP-Score). While low risk tumors were defined by SATB1 positivity (high SATB1 expression in at least one tissue core), genomic stability and a GS ≤ 6, high risk patients were either SATB1 negative or showed genomic instability or had a GS ≥ 7. From the present collective 47 patients could be stratified using this new PCP-Score, of whom 30 were assigned as high risk and 17 as low risk tumors. Patients with high risk tumors showed a significant shorter OS of 11.7 years (CI, 9.4–14) compared to 19 years (CI, 16.9–21.1) for low risk patients (*p* = 0.001, Fig. 2B) and a fourfold increased risk of dying in age adjusted univariable Cox regression analysis (age-adjusted HR 4, CI 1.701–9.407, *p* = 0.001, Table S1). Hazard ratios for the risk factors and their combinations are shown in Fig. 3. Indeed, this new score was the strongest independent predictor for OS in a multivariable model including the established prognostic factors tumor stage, preoperative PSA, Gleason Score and combinations of prognostic factors that were significant in univariable Cox regression (HR 3.277, CI 1.182–9.087, *p* = 0.023). In order to ascertain the contribution of genomic instability to the predictive value of the new score, we fitted a model with and without genomic instability yielding a noticeable gain in the score when including genomic instability (Table S3). The statistical performance decreased when immunopositivity was defined by ≥ 2 tissue cores with high SATB1 expression (*p* = 0.010, Table S1, Fig. 3).

When considering only patients with ≤ 1 tissue core with high SATB1 expression (n = 19), the age adjusted HR for the PCP-Score was 5.346 (*p* = 0.036, CI 1.112–25.695) whereas it was 3.045 (*p* = 0.042, CI 1.042–8.902) for patients with ≥ 2 tissue cores with high SATB1 expression. Notably, grouping patients according to GS, genomic instability and SATB1 expression in one randomly chosen tissue core was not predictive for OS (Fig. 3).

## Discussion

An accurate estimation of possible tumor progression and survival is of unmet clinical need in order to offer individualized therapy. In this context, differences in protein expression (SATB1, SPIN1, TBB5, VIME, TPM4) between benign prostate tissue and prostate cancer with respect to clinicopathological risk factors, tumor heterogeneity and genomic instability were assessed. In publicly available databases (proteinatlas.org) the expression of SATB1, TBB5 and VIME is stated as being moderate to weak in prostate cancer and for SPIN1 and TPM4 as strong to weak depending on the antibody used^31^. These results are generally comparable to ours, however, due to missing information on tumor grade and clinical parameters in the human protein atlas a validation of our results is not fully possible.

Evaluating aneuploidy has emerged as a means to predict aggressiveness and the clinical course of (prostate) cancer^32–34^. We could show that tumor aneuploidy reflecting genomic instability was strongly associated with a GS ≥ 7 indicating a higher frequency in dedifferentiated prostate cancer tissue with worse prognosis. Furthermore, we found a significant association of genomic instability and a decreased OS. This is in line with findings that aneuploid radical prostatectomy specimens show a strong correlation with higher tumor grade and shorter recurrence free survival^35, 36^. Nevertheless, detection of aneuploidy in one randomly chosen tissue section of a tumor is not predictive for disease recurrence whereas multiple sampling denoted aneuploidy as a prognostic factor^8^. In this context, it is important that underestimation of the tumor’s fate due to tumor heterogeneity is not only minimized by taking multiple samples but also by combining the assessment of genomic instability and GS^8^. This is consistent with our results showing that not a single parameter alone but the combination of GS, SATB1 expression, and genomic instability has the strongest predictive power.

While TBB5, TPM4 and VIME only showed differential expression between normal and cancerous tissue, high expression of SATB1 and SPIN1 was also significantly associated with good prognosis.

SATB1 is a global chromatin organizer that regulates gene expression over long distances by chromatin looping and recruitment of chromatin remodeling enzymes^16, 37^. We found it to be elevated in prostate cancer tissue compared to benign tissue and associated with longer OS for patients with high expression. These results were in accordance with the human protein atlas, where immunohistochemical SATB1 expression is reported as negative to weak in normal prostate tissue and as weak to moderate in prostate cancer, though higher than in normal tissue^31^. We could therefore achieve comparable results as this publicly available database which reflects the representativity of our study cohort and methods. Additionally, the proteinatlas.org indicates that the loss of SATB1 or weak expression in tumor tissue was associated with worse overall survival for renal cell carcinoma and pancreatic cancer, while a higher expression is associated with longer overall survival^31^. These results also coincide with our findings concerning prostate cancer. However, our results were in contrast to other studies in which SATB1 overexpression correlated to aggressive tumor biology and metastasis in prostate cancer^12, 20, 38^. SATB1 has been suggested to promote tumor growth and metastasis by upregulation of MMP2 and Vimentin with simultaneous downregulation of E-Cadherin as hallmarks for EMT^39, 40^. However, and in accordance with our results, increased SATB1 protein levels also correlated with a better prognosis in lung and colorectal cancer^41, 42^. Furthermore, there is evidence that SATB1 overexpression did not promote breast cancer progression and was associated with a benefit in disease-free survival in estrogen receptor positive breast cancer patients^43, 44^.

SATB1 is a highly phosphorylated and acetylated protein^45^ so that controversial expression differences may be explained by posttranslational modifications^46^. Additionally, SATB1 molecules showed a wide spatial variation in their distribution in thymocyte cell nuclei, indicating that the majority of SATB1 molecules are highly dynamic with varying ability to access different regions of the nucleus^47^. Super-resolution stimulated emission depletion (STED) imaging might identify distinct spatial distribution patterns of SATB1 in tumor cell nuclei which could further explain its function and prognostic value^48^. However, the present study suggests a positive SATB1 expression when this expression was detected in the cytoplasm and/or the cell nucleus of glandular tumor cells which was in accordance with the human protein atlas^31^.

Additionally, well differentiated proliferating cancer cells might express SATB1 in contrast to benign tissue or poorly differentiated, aneuploid cancer cells. Especially the loss of SATB1 could explain a deregulated chromatin organization and thus favor aneuploid, genomically instable cell populations.

Furthermore, prostate cancer is a highly heterogenic disease characterized by different molecular traits across different cell populations within one tumor^49^. These facts coincide with the frequent incidence of genomic instability and the high degree of variability in the protein expression of SATB1 found in this study. Only one single biopsy might therefore neither be representative nor sufficient for diagnostics^8^. This prompted us to evaluate the prognostic value of SATB1 by choosing either one randomly chosen tissue core, counting if at least one of four tissue cores shows high SATB1 expression, or using the number of tissue cores with high SATB1 expression. Particularly, we could show that the expression of SATB1 in only one randomly chosen tissue core cannot predict OS, whereas counting the number of tissue cores with a high SATB1 expression or defining a tumor as SATB1 positive when at least one of four tissue cores per patient showed a high SATB1 expression could reveal SATB1 as an predictor for longer OS. Therefore, our results support a multiple sampling strategy to address the diagnostic challenge of intra-tumor heterogeneity.

Most notably, the parameters SATB1 (when assessed by multiple samples from different tumor sites), pathological GS and genomic instability could be combined as a new Prostate Cancer Prediction Score (PCP-Score) which is a significant predictor for OS. By differentiating high risk patients from low risk patients with the novel PCP-Score, long term survivors with a mean survival of 19 years could be separated from patients with shorter postoperative survival of 11.7 years after radical prostatectomy. Interestingly, grouping our patients based on D’Amico’s classification for risk of PSA recurrence showed no significant association with overall survival.

However, limitations of our study are the small sample size and that we used stored cancer tissue of a retrospective cohort after radical prostatectomy without information on disease free survival. It has also to be noted though that the patients evaluated here had a mean age of 63 years which is comparably younger compared to the average age of 72 years for the occurrence of prostate cancer^50^. While we are not aware of any potential bias, we cannot exclude that our results might be specifically benefitting a younger age group.

Although this PCP-Score was built on postoperative data after radical prostatectomy, it will likely also predict disease progression in preoperative settings: For SATB1 the pretreatment situation was simulated by taking four TMA cores of representative tumor areas comparable to needle biopsies at initial diagnosis. Moreover, a good correlation between the ploidy status of preoperative needle biopsy and postoperative radical prostatectomy specimen was shown^51^. We assume that especially in preoperative biopsy specimens with an underestimated GS^7^, also assessing SATB1 expression and the degree of genomic instability could be of high value in predicting clinical outcome. However, for implementation of the new Score in preoperative routine diagnostics, large validation studies using external datasets and patient material from preoperative biopsies would be necessary.

In conclusion, we demonstrated that our Score containing SATB1 expression, examined by immunohistochemical analysis in a multiple sample approach, combined with genomic instability and GS is a predictor for prognosis in prostate cancer potentially outperforming risk stratifications in current use. Further multi-center validation experiments including the evaluation of disease-free survival are warranted.

## Supplementary Information

Supplementary Information.

## Data Availability

The datasets used and/or analyzed during the current study are available from the corresponding author on reasonable request.

## Abbreviations

AEC: Aminoethylcarbazole
CI: Confidence Interval
DAB: Diaminobencidine
EMT: Epithelial-Mesenchymal-Transition
GS: Gleason Score
HE: Hematoxylin and Eosin
IRS: Immunoreactive Score
OS: Overall Survival
PCP-Score: **P**rostate **C**ancer **P**rediction Score
PSA: Prostate-Specific-Antigen
SATB1: Special-AT-rich-binding-Protein-1
SPIN1: Spindlin-1
TBB5: β-Tubulin
TPM4: Tropomyosin-4
TMA: Tissue-Microarray
VIME: Vimentin
