# Supplementary material for: SATB1, genomic instability and Gleason grading constitute a novel risk score for prostate cancer
Source: Sci Rep. 2021 Dec 27;11:24446. doi: 10.1038/s41598-021-03702-0 (PMC8712510; doi:10.1038/s41598-021-03702-0)
Supplement: Supplementary file 1 — Supplementary Information. [file 41598_2021_3702_MOESM1_ESM.docx]

**Supplemental Material**

**Supplemental Data 1**

Histograms were classified according to Auer.^28^ Histograms with one single peak in the diploid or near diploid region (1,5-2,5c) were classified as Type I. Type II histograms were defined by a single peak in the tetraploid region (3,5-4,5c), or peaks in both diploid and tetraploid region. Type III histograms were characterized by DNA values ranging between diploid and tetraploid region with only a few cells exceeding DNA values of 5c (<5%). Scattered DNA values with >5% of the cells exceeding the 5c region were defined as Type IV histograms which represent aneuploid cell populations with decreased genomic stability. Thus, tumors showing a DNA distribution pattern corresponding to type I-III histograms were assessed as genomically stable, whereas those showing a DNA distribution pattern according to type IV histograms were classified as genomically instable.

**Supplemental Data 2**

TMA sections were deparaffinized by Xylol and rehydrated in a graded series of ethanol, followed by heating in retrieval buffer using a microwave oven. Subsequently, sections were blocked with 3% hydrogen peroxide in methanol, incubated in 0.1% Triton-X-100 in PBS, and treated with horse (Vectastain^®^ Elite ABC Kit Universal, VECTOR Laboratories Inc., Burlingame, Ca, USA) or goat serum (Dako Cytomation A/S, Glostrup, Denmark) in PBS (pH 7.4). Primary antibodies against anti-VIME (dilution 1:200, Sigma-Aldrich, St. Louis, USA, #V6389), monoclonal anti-TBB5 (dilution 1:600, Sigma-Aldrich, St. Louis, USA, #T4026), monoclonal anti-TPM4 (dilution 1:700, Abcam^®^ plc, Cambridge, UK, # ab58332), polyclonal anti-SPIN1 (dilution 1:300, Abcam^®^ plc, Cambridge, UK, # ab118784) and anti-SATB1 (dilution 1:50, kindly provided by R. Klooster, University of Leiden, Netherlands) diluted in antibody diluent (Antibody Diluent with Background Reducing Components, Dako Cytomation A/S, Glostrup, Denmark) were incubated overnight at 4°C. Anti-SATB1 is a single

domain VHH-antibody fragment consisting of one single N-terminal domain without Fc part and offers the advantage of binding to small, hidden epitopes with excellent penetration of tissue.^49^ The specificity of this VHH-antibody fragment (clone 2D2) was previously confirmed by ELISA, cell staining as well as Western Blot and immunoprecipitation at Leiden University Medical Center, Netherlands. Furthermore, no cross-reactivity with recombinant SATB2 was observed.

Subsequently, all TMA sections were incubated with biotin labelled secondary antibodies (Vectastain^®^ Elite ABC Kit Universal, VECTOR Laboratories Inc., Burlingame, Ca, USA). For SATB1, incubation was carried out with Mouse-anti VSV secondary antibody (kindly provided by R. Klooster, University of Leiden, Netherlands) followed by a biotinylated tertiary antibody (biotinylated goat-anti-mouse, VECTOR Laboratories Inc., Burlingame, Ca, USA, # BA-9200). A combination of avidin-peroxidase-complex-solution and diaminobencidine (DAB, Liquid DAB + Substrate Chromogen System, Dako Cytomation A/S, Glostrup, Denmark) or aminoethylcarbazole (AEC, AEC substrate chromogen Ready-to-Use, Dako Cytomation A/S, Glostrup, Denmark) was used as chromogen. All TMA-sections were counterstained with Hematoxylin and coverslipped with Aquatex^©^ (Merck KGaA, Darmstadt). For further investigation the slides were scanned and digitized with a 3D Panoramic DESK^®^ Scanner (3DHISTECH Ltd. Budapest, Hungary).

After immunohistochemical staining, scoring was performed semiquantitatively by one independent senior pathologist (A.G.) according to the immunoreactive Score (IRS)^50^ (Figure S2-5). For VIME, an alternative score regarding the percentage of positive tumor cells was used: Score 0 - negative staining of all tumor cells; Score 1 - up to 20% stained tumor cells; Score 2 - 21 to 50 % stained tumor cells; Score 3 - more than 50% stained tumor cells (Figure S6). A high expression was defined as IRS 6-12 for SATB1 and TPM4, and as IRS 9-12 for SPIN1 and TBB5. For VIME a tissue core was assessed for high expression when showing a Score of 1 - 3.


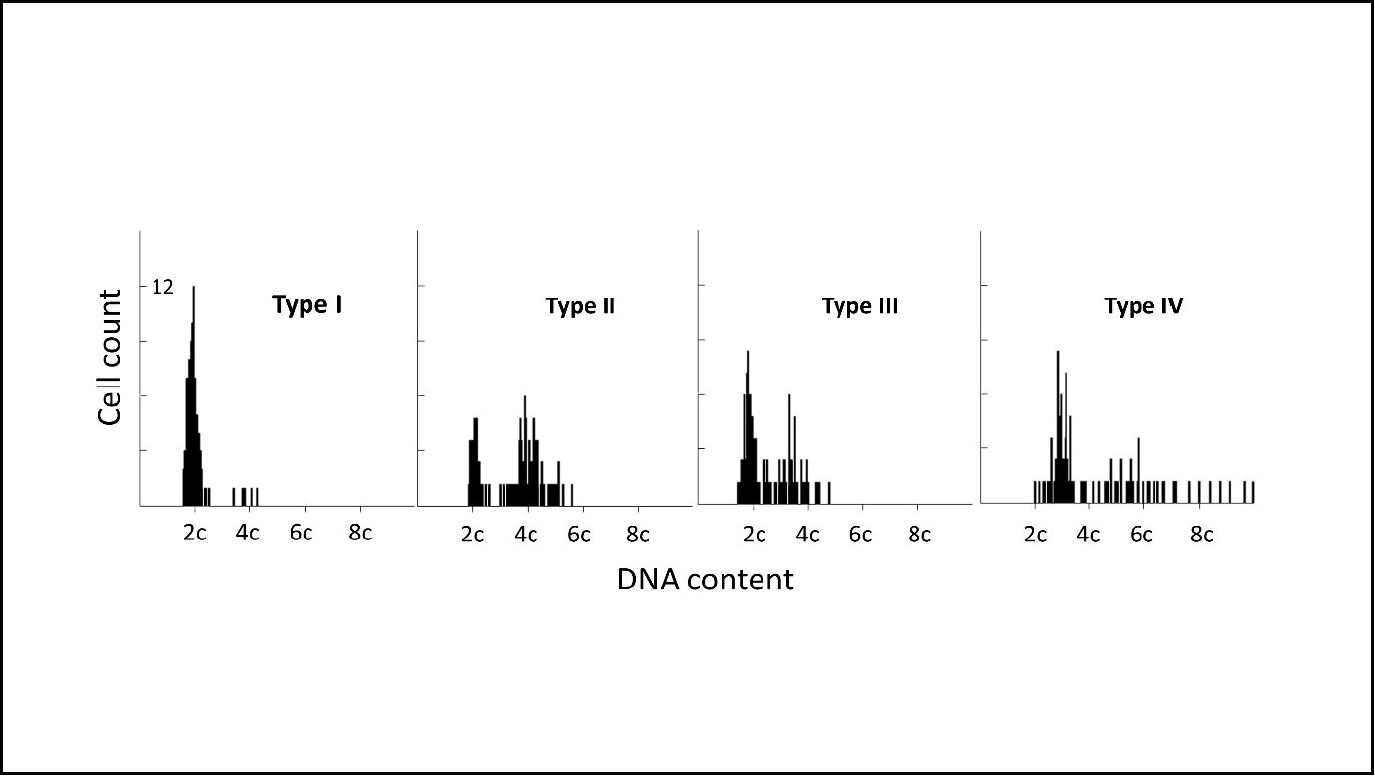


**Figure S1**

DNA profiles from four different patients of the study collective. Type I histogram with DNA values in the range of 2c (diploid). Type II with well-defined peaks in the range 2c and 4c (tetraploid). Type III with slightly displaced peaks near 2c and 4c and <5% of the tumor cells exceeding 5c (diploid proliferative). Type IV histograms with scattered DNA values and >5% of the tumor cells exceeding 5c (genomically instable).


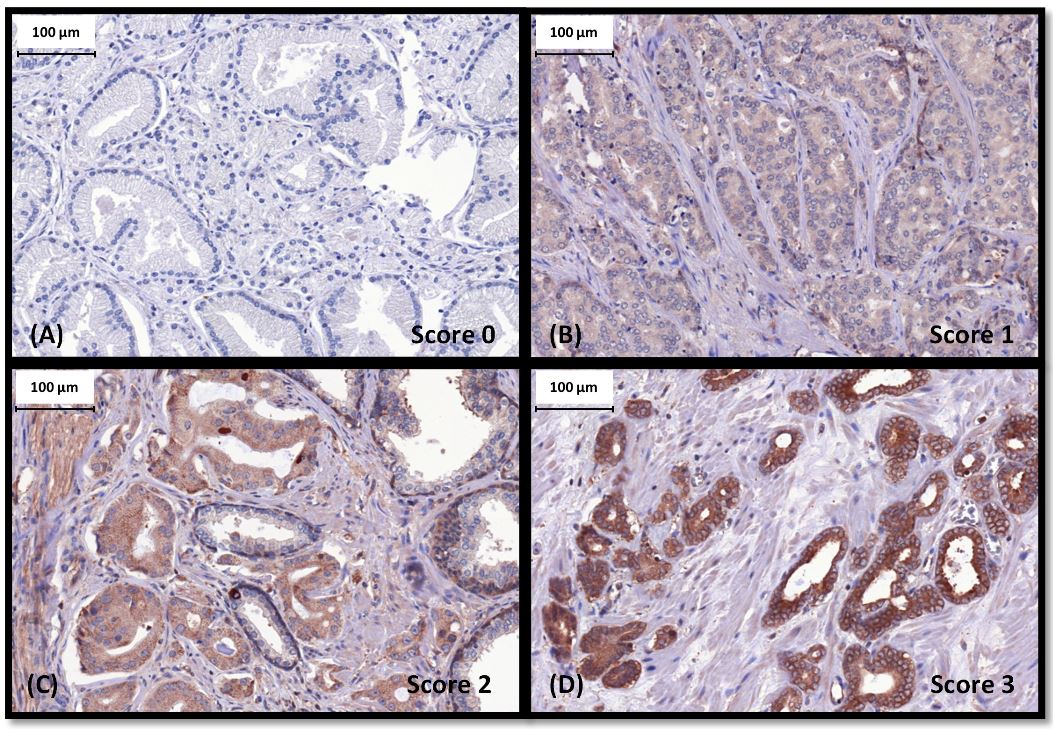


**Figure S2**

Immunhistochemical staining of SATB1 and semiquantitative scoring according to IRS. **(A)** Score 0 (IRS: 0-2). **(B)** Score 1 (IRS: 3-4). **(C)** Score 2 (IRS: 6-8). **(D)** Score 3 (IRS: 9-12). Brown: Diaminobencidine stained cells. Blue: Counterstaining with Hematoxylin. Magnification 40x.


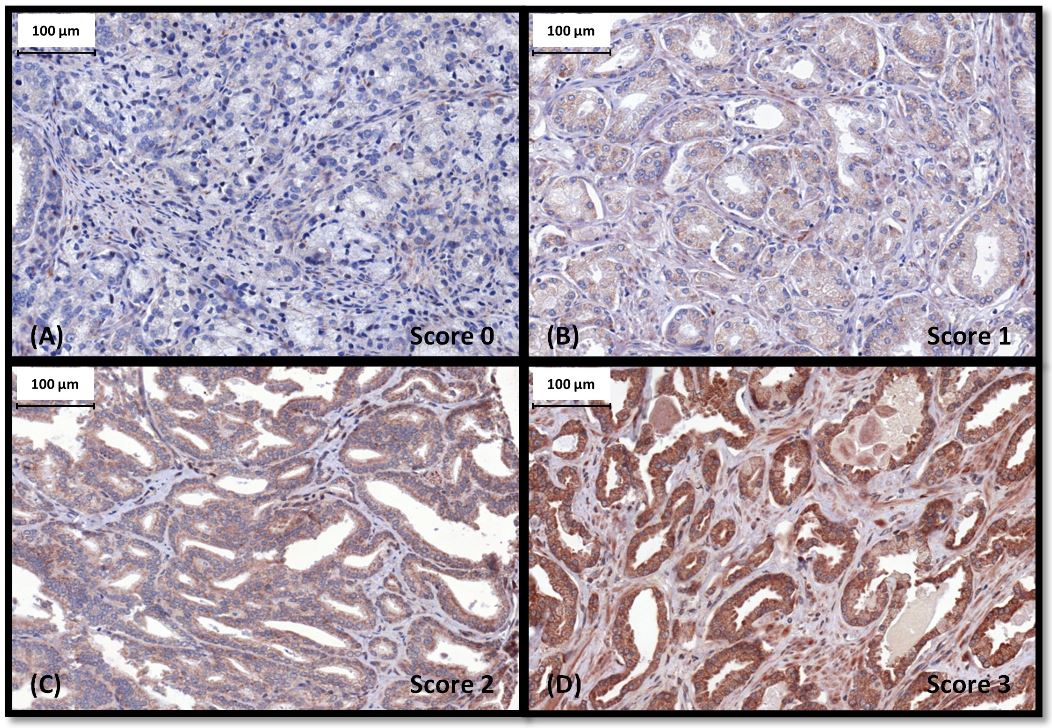


**Figure S3**

Immunhistochemical staining of SPIN1 and semiquantitative scoring according to IRS. **(A)** Score 0 (IRS: 0-2). **(B)** Score 1 (IRS: 3-4). **(C)** Score 2 (IRS: 6-8). **(D)** Score 3 (IRS: 9-12). Brown: diaminobencidine stained cells. Blue: Counterstaining with Hematoxylin. Magnification 40x.


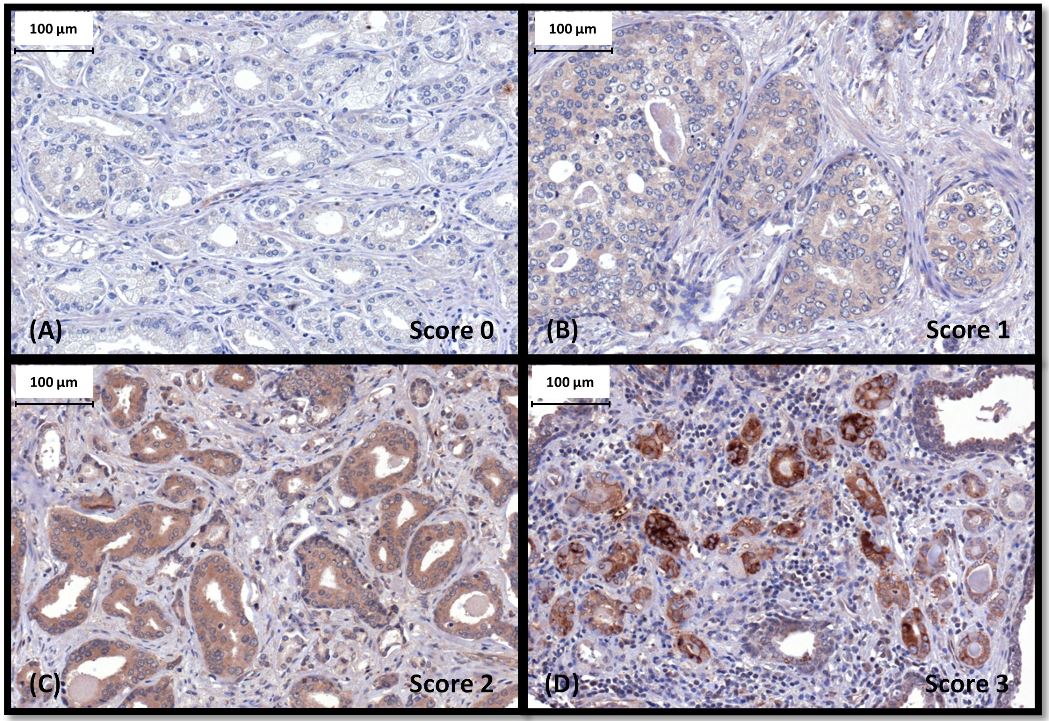


**Figure S4**

Immunhistochemical staining of TPM4 and semiquantitative scoring according to IRS. **(A)** Score 0 (IRS: 0-2). **(B)** Score 1 (IRS: 3-4). **(C)** Score 2 (IRS: 6-8). **(D)** Score 3 (IRS: 9-12). Brown: diaminobencidine stained cells. Blue: Counterstaining with Hematoxylin. Magnification 40x.


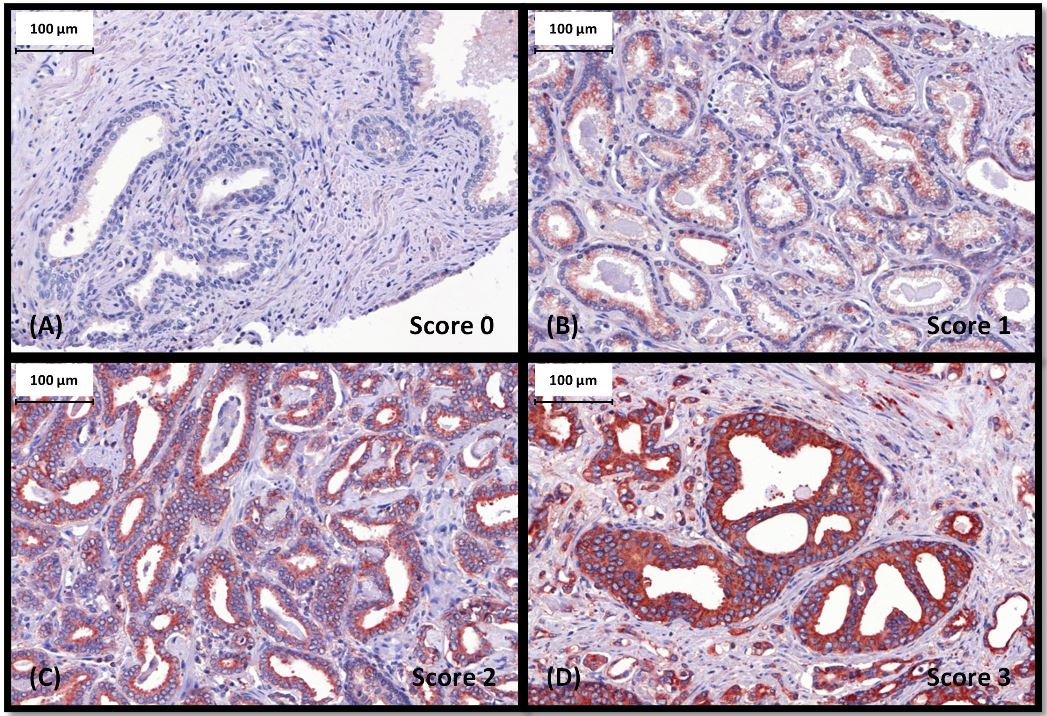


**Figure S5**

Immunhistochemical staining of TBB5 and semiquantitative scoring according to IRS. **(A)** Score 0 (IRS: 0-2). **(B)** Score 1 (IRS: 3-4). **(C)** Score 2 (IRS: 6-8). **(D)** Score 3 (IRS: 9-12). Red: aminoethylcarbazole stained cells. Blue: Counterstaining with Hematoxylin. Magnification 40x.


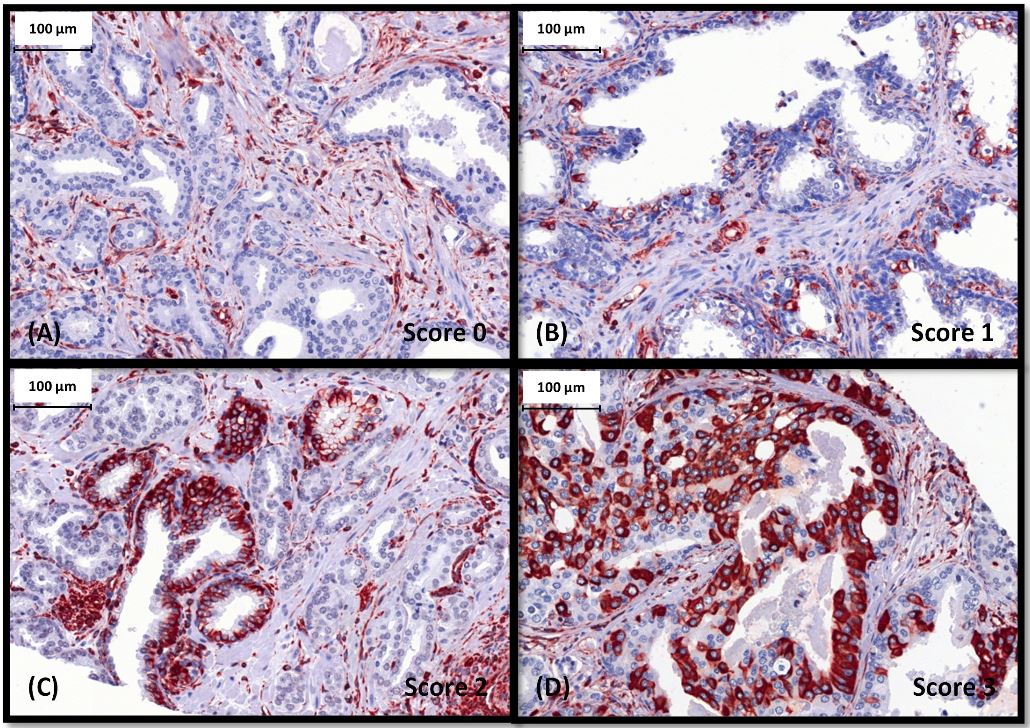


**Figure S6**

Immunhistochemical staining of VIME and semiquantitative scoring according to percentage of positive stained tumor cells. **(A)** Score 0 – negative staining of all tumor cells **(B)** Score 1 – up to 20% stained tumor cells. **(C)** Score 2 – 21 to 50% stained tumor cells. **(D)** Score 3 – more than 50% stained tumor cells. Red: aminoethylcarbazole stained cells. Blue: Counterstaining with Hematoxylin. Magnification 40x.


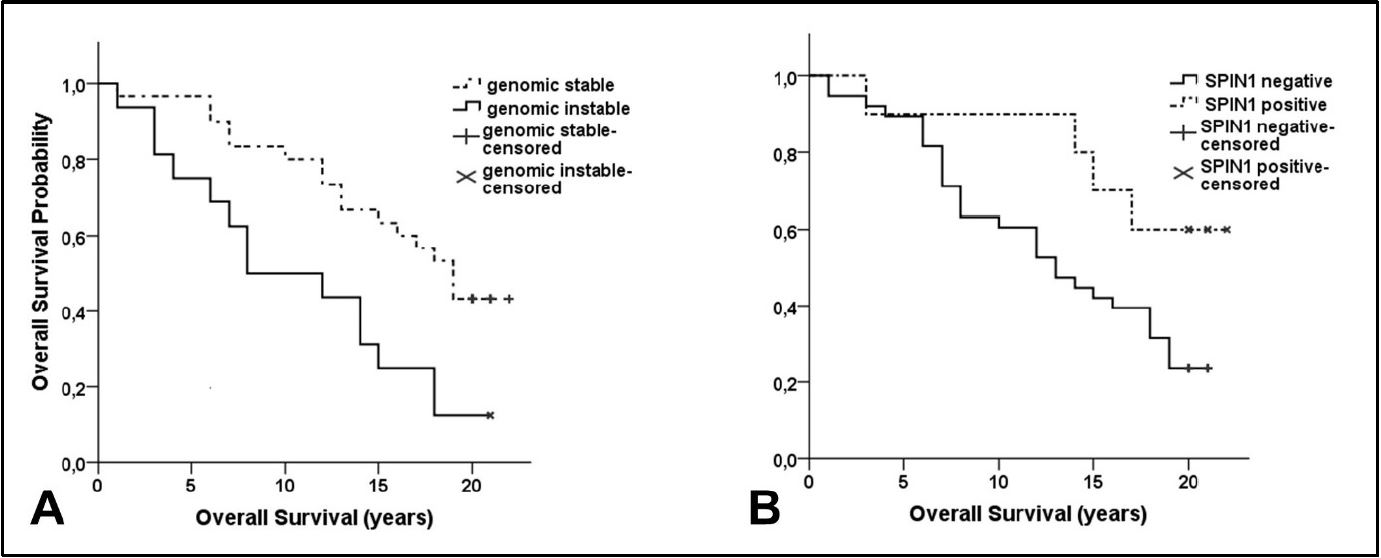


**Figure S7**

Overall Survival stratified by: **(A)** Genomic stability, p = 0.006. **(B)** SPIN1 positivity, p = 0.048. P-values for the log-rank test.

| **Variable** | Mean time to death [years] (95% CI) | log-rank | **Age adjusted Cox Regression Analysis** | | |
| --- | --- | --- | --- | --- | --- |
|  |  | p-Value | Hazard Ratio | 95% CI | p-Value |
| **Tumor stage**  ≤ T2b  T3-T4 | 16.9 (13.7 to 20.2)  13.4 (11.1 to 15.7) | 0.180 | 1.923 | 0.822 to 4.496 | 0.131 |
| **PSA**  ≤ 10  > 10 | 15.9 (12.7 to 19)  13.5 (10.7 to 16.3) | 0.449 | 1.819 | 0.735 to 4.499 | 0.195 |
| **GS**  4-6  ≥ 7 | 17.9 (15.9 to 20)  11.0 (8.5 to 13.6) | 0.002 | 3.394 | 1.615 to 7.132 | **0.001** |
| **Ploidy status**  Genomic stability,  Genomic instability, | 16.5 (14.3 to 18.8)  10.8 (7.6 to 14) | 0.006 | 2.672 | 1.294 to 5.515 | **0.008** |
| **SATB1**  positive  negative | 15.5 (13.3 to 17.6)  10.3 (6.5 to 14) | 0.020 | 0.413 | 0.192 to 0.886 | **0.023** |
| **SATB1 (number of tissue cores)**  Expression in ≥2 cores high  Expression in≤1 core high | 16.3 (14.1 to 18.5)  11.1 (8.0 to 14.2) | 0.018 | 0.479 | 0.238 to 0.965 | **0.039** |
| **GS, ploidy status**  GS ≤6 and genomically stable  GS ≥7 or genomically instable | 18.5 (16.5 to 20.5)  11.6 (9.0 to 14.2) | 0.003 | 3.356 | 1.520 to 7.413 | **0.003** |
| **GS, SATB1**  GS ≤6 and SATB1 positive  GS ≥7 or SATB1 negative | 18.3 (16.1 to 20.5)  11.3 (8.9 to 13.7) | 0.001 | 3.785 | 1.726 to 8.303 | **0.001** |
| **GS, SATB1 (number of tissue cores)**  GS ≤6 and SATB1 in ≥2 cores high  GS ≥7 or SATB1 in ≤1 core high | 18.2 (15.7 to 20.8)  12.1 (9.8 to 14.4) | 0.005 | 3.092 | 1.329 to 7.194 | **0.009** |
| **Ploidy status, SATB1**  Genomically stable and SATB1 positive  Genomically instable or SATB1 negative | 17.1 (14.8 to 19.3)  11.2 (8.3 to 14.1) | 0.005 | 2.761 | 1.335 to 5.710 | **0.006** |
| **Genomic stability, SATB1 (number of tissue cores)**  Genomically stable and SATB1 high in ≥2 cores  Genomically instable or SATB1 high in ≤1 core | 17.3 (14.8 to 19.7)  12.1 (9.5 to 14.8) | 0.019 | 2.286 | 1.082 to 4.829 | **0.030** |
| **GS, genomic stability, SATB1 (number of tissue cores)**  low risk (GS ≤6 and genomically stable and SATB1 high in ≥2 tissue cores)  high risk (GS ≥7 or genomically instable or SATB1 high in≤1 tissue cores) | 19.2 (16.7 to 21.6)  12.4 (10.2 to 14.6) | 0.005 | 3.531 | 1.347 to 9.258 | **0.010** |
| **PCP-Score**  low risk (GS ≤6 and genomically stable and SATB1 positive)  high risk (GS ≥7 or genomically instable or SATB1 negative) | 19.0 (16.9 to 21.1)  11.7 (9.4 to 14.0) | 0.001 | 4.000 | 1.701 to 9.407 | **0.001** |

**Table S1:** Survival Analysis. SATB1 positive = at least one tissue core with a high protein expression. SATB1 ≥2 cores = ≥2 tissue cores with a high protein expression. SATB1 ≤ 1 core = ≤1 tissue core with a high SATB1 expression.

**Table S2:** Significant associations between

clinicopathological parameters and ploidy status.

| **Parameter** | **DNA ploidy status** | | | |
| --- | --- | --- | --- | --- |
|  | **euploid,**  **genomically stable**  **(n = 32)** | **aneuploid, genomically instable**  **(n = 16)** | N | p-value* |
| **Gleason Score**  4-6  7-10 | 21 (91.3%)  11 (44%) | 2 (8.7%)  14 (56%) | 48 | **0.001** |
| **Tumor stage**  ≤ T2b  ≥T3 | 8 (61.5%)  24 (70.6%) | 5 (38.5%)  10 (29.4%) | 47 | 0.728 |
| **PSA-value (ng/ml)**  ≤ 10  > 10 | 10 (71.4%)  14 (58.3%) | 4 (28.6%)  10 (41.7%) | 38 | 0.501 |
| * P-value for Fisher’s Exact Test; significant p- values are highlighted in bold. | | | | |

| **Model with genomic instability** | **Cox Regression Analysis** | | |
| --- | --- | --- | --- |
|  | Hazard Ratio | 95% CI | p-Value |
| Number of tissue cores with high SATB1 expression  GS(≥7) or genomic instability or SATB1 negative | 0.500  **3.354** | 0.246 to 1.018  **1.422 to 7.914** | 0.056  **0.006** |
| **Model without genomic instability** |  | | |
| Number of tissue cores with high SATB1 expression  GS(≥7) or SATB1 negative | 0.576  3.051 | 0.284 to 1.171  1.376 to 6.767 | 0.128  0.006 |

**Table S3:** Model with and without genomic instability.
